# An overview of methods using ^13^C for improved compound identification in metabolomics and natural products

**DOI:** 10.3389/fpls.2015.00611

**Published:** 2015-08-25

**Authors:** Chaevien S. Clendinen, Gregory S. Stupp, Ramadan Ajredini, Brittany Lee-McMullen, Chris Beecher, Arthur S. Edison

**Affiliations:** ^1^Southeast Center for Integrated Metabolomics, University of Florida, Gainesville, FL, USA; ^2^Department of Biochemistry and Molecular Biology, University of Florida, Gainesville, FL, USA; ^3^The Scripps Research Institute, La Jolla, CA, USA; ^4^IROA Technologies, Ann Arbor, MI, USA

**Keywords:** isotope, NMR, LC-MS, metabolomics, natural products

## Abstract

Compound identification is a major bottleneck in metabolomics studies. In nuclear magnetic resonance (NMR) investigations, resonance overlap often hinders unambiguous database matching or de novo compound identification. In liquid chromatography-mass spectrometry (LC-MS), discriminating between biological signals and background artifacts and reliable determination of molecular formulae are not always straightforward. We have designed and implemented several NMR and LC-MS approaches that utilize ^13^C, either enriched or at natural abundance, in metabolomics applications. For LC-MS applications, we describe a technique called isotopic ratio outlier analysis (IROA), which utilizes samples that are isotopically labeled with 5% (test) and 95% (control) ^13^C. This labeling strategy leads to characteristic isotopic patterns that allow the differentiation of biological signals from artifacts and yield the exact number of carbons, significantly reducing possible molecular formulae. The relative abundance between the test and control samples for every IROA feature can be determined simply by integrating the peaks that arise from the 5 and 95% channels. For NMR applications, we describe two ^13^C-based approaches. For samples at natural abundance, we have developed a workflow to obtain ^13^C–^13^C and ^13^C–^1^H statistical correlations using 1D ^13^C and ^1^H NMR spectra. For samples that can be isotopically labeled, we describe another NMR approach to obtain direct ^13^C–^13^C spectroscopic correlations. These methods both provide extensive information about the carbon framework of compounds in the mixture for either database matching or *de novo* compound identification. We also discuss strategies in which ^13^C NMR can be used to identify unknown compounds from IROA experiments. By combining technologies with the same samples, we can identify important biomarkers and corresponding metabolites of interest.

## Introduction

Metabolomics and natural product studies share many common goals. Indeed, we and others have argued that the two fields are essentially the same (Robinette et al., 2012). Both have the ultimate goal of identifying a small molecule that is responsible for a particular activity or phenotype. Both utilize the same analytical tools, namely mass spectrometry (MS) and nuclear magnetic resonance (NMR). However, metabolomics and natural products traditionally approach the identification task from different directions. Whereas metabolomics utilizes less chemical purification and more statistical analysis, natural products studies generally utilize some sort of biological assay to guide the purification and identification of an active compound, which may be biosynthesized from any metabolic pathway(s). We believe that by applying the most powerful tools from each field, the task of compound identification can be greatly simplified.

In our experience, the use of ^13^C isotopes (^13^C) in metabolomics studies greatly enhances the ability to identify and quantify biomarkers. NMR and MS, commonly used in natural products studies, can be efficiently applied to complex metabolic mixtures through the simplification of spectra by ^13^C filtering. This concept is not new, as ^13^C has a long history in targeted metabolomics (Sumner et al., 1992, 2003; Fennell et al., 2006; Garner et al., 2006; Kwon et al., 2014), flux studies (Merritt et al., 2007; Moseley et al., 2011; Moreno et al., 2014; Purmal et al., 2014; Yang et al., 2014; Buescher et al., 2015), and *in vivo* metabolic studies (Golman et al., 2006; Schroeder et al., 2008; Colombo Serra et al., 2012). However, ^13^C has not seen widespread use in untargeted metabolomics, where we believe it has great potential to improve compound identification.

As we will demonstrate below, ^13^C can be utilized in liquid chromatography-MS (LC-MS) studies to allow for the discrimination between biosynthesized metabolites and background noise, which is a common problem in LC-MS. Moreover, through such ^13^C labeling strategies, the number of carbons of each metabolite can be determined, greatly enhancing the determination of molecular formulae. The same labeling strategy can be used to obtain accurate relative quantification of metabolites in an untargeted LC-MS experiment, which can be difficult to quantify without the use of internal standards (Bennett et al., 2008; Feldberg et al., 2009; Giavalisco et al., 2009; Bueschl et al., 2014). In NMR studies, ^13^C can also provide several advantages. Perhaps most important is the large chemical shift range (∼200 ppm) of ^13^C compared to ^1^H (∼10 ppm). This allows for less overlap in NMR spectra and for more efficient statistical analysis. ^13^C chemical shifts alone, or in addition to ^1^H chemical shifts, allow for more efficient database matching for compound identification or dereplication. Finally, direct ^13^C correlations that can be obtained from NMR studies are an extremely effective way to determine the identity of unknown metabolites or ones that are not in databases.

With all of these advantages of ^13^C, why is it not more commonly used in NMR? The most obvious answer is the low isotopic abundance of ^13^C (1.1%). This effectively dilutes the signal of interest by 100-fold from standard ^1^H-based NMR methods. More importantly, the 1.1% abundance of ^13^C leads to low probabilities of two or more ^13^C atoms being next to each other in the same molecule, which is a necessary condition for many of the approaches we will describe below. All of these problems can be offset by isotopically labeling with ^13^C. In some cases, isotopic labeling is simple and cost effective, while in others it is difficult or impossible. We also present some strategies below to get around the problem of labeling.

The plan of this review is as follows: First we will describe an LC-MS based method called isotopic ratio outlier analysis (IROA; de Jong and Beecher, 2012) and show how this technique can achieve many of the advantages described above (Stupp et al., 2013). Next, we will show how ^13^C can be used at natural abundance in NMR metabolomics studies (Clendinen et al., 2014). This relatively simple approach can provide much more robust compound identification through database matching than by using ^1^H NMR alone. We will then describe a method using ^13^C enrichment that utilizes the 2D NMR experiment called INADEQUATE [incredible natural abundance double quantum transfer experiment (INADEQUATE); Clendinen et al., 2015]. Although INADEQUATE was developed for samples at natural abundance ^13^C, we use the same pulse sequence with ^13^C-labeled samples and thus keep the same name to avoid confusion about which NMR experiment is being used. INADEQUATE is perhaps the “gold standard” for natural product identification by NMR, and it has great advantages in metabolomics studies. Finally, we describe how ^13^C NMR can be used to identify unknown metabolites from an IROA LC-MS experiment.

The majority of applications we use to illustrate the ^13^C methods focus on the rich-soil or compost-dwelling nematode, *Caenorhabditis elegans*. All of the methods we present can be applied to plants or, if they can be cultured, plant parasitic nematodes. The NMR technique using natural abundance ^13^C NMR can use any type of sample if enough material is available. Other methods may require ^13^C isotopic labeling, which is easily done in plants. Some interesting recent applications of whole plant labeling include: (1) the examination of carbon flux in isoprenoid pathways in poplar grown with ^13^CO_2_ (Ghirardo et al., 2014), (2) the identification of sulfur-containing metabolites from onions using high-resolution FT-ICR MS (Nakabayashi et al., 2013), (3) the ^13^C isotopic labeling of tomatoes (Moran et al., 2013) and parsley, spinach, and peppermint (Gleichenhagen et al., 2013) to obtain biologically active phytochemicals for human metabolic studies, and (4) the ^13^CO_2_ labeling of potato plants to identify metabolites that are released by their roots and are subsequently incorporated into fungi in the rhizosphere (Hannula et al., 2012).

## Isotopic Ratio Outlier Analysis

**FIGURE 1:**
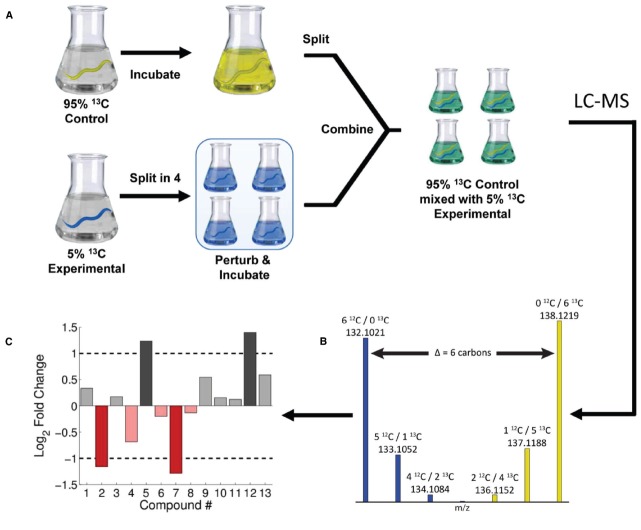
**Summary of IROA methods using ***C. elegans***.** Control and experimental populations of *C. elegans* were grown on 95 and 5% ^13^C labeled bacteria, respectively **(A)**. The experimental population were split prior to perturbation and then combined with control yielding mixed 95 and 5% ^13^C populations. LC-MS analysis on IROA samples reveals isotopic peak patters that provide carbon number **(B)** as well as relative quantitation **(C)**. Figure used with permission from Stupp et al. (2013).

Isotopic ratio outlier analysis is an LC-MS-based stable isotope labeling strategy that allows for the discrimination of real compounds from artifacts (de Jong and Beecher, 2012; Stupp et al., 2013). In an IROA experiment, a group of experimental cells, tissues, or organisms, are labeled with 5% ^13^C, while a common reference or control population is labeled with 95% ^13^C. Extracts from both samples are mixed together, ideally in a 1:1 ratio so that the 5% labeled material is mixed with a comparable amount of the 95%. The mixed sample is then extracted and analyzed by LC-MS. A summary of this method is provided in Figure 1, using *Caenorhabditis elegans* as an example. Mixing the extracts reduces the technical variation between experimental and control pairs, because the 5 and 95% extracts are run at the same time on the LC-MS. In addition, the 95 and 5% gives rise to a distinctive isotopic peak pattern, allowing for simple discrimination between biosynthesized compounds and noise. An additional benefit of mixing is the decrease in the total number of samples to be run on the spectrometer, reducing the LC-MS cost per sample by half.

**FIGURE 2:**
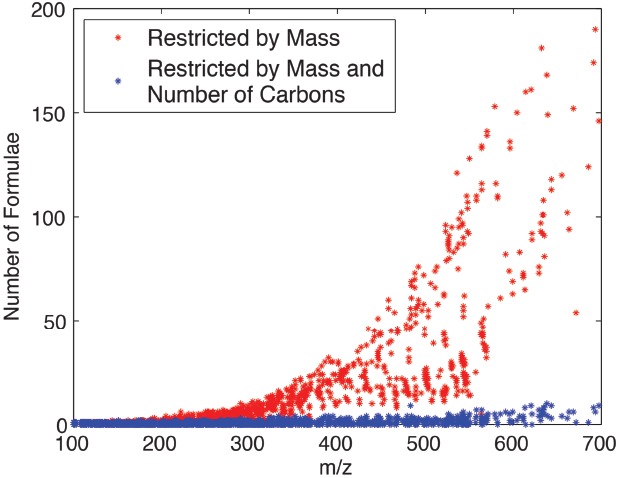
**Number of possible molecular formulae as a function of mass restricted by mass alone (red) or both mass and number of carbons (blue).** When restrained by both mass and number of carbons, the number of possible molecular formulae for a given mass is severely restricted. Figure adapted with permission from Stupp et al. (2013).

Unlike methods that use full isotopic labeling (>99% ^13^C) vs. unlabeled or natural abundance (∼1.1% ^13^C; Birkemeyer et al., 2005; Bueschl et al., 2014; Huang et al., 2014; Neumann et al., 2014), the use of 5% and the 95% ^13^C facilitates the detection of isotopic peaks that may be absent using other methods. This is because at natural abundance, particularly for low intensity features, more than one isotopic peak is not always detectable or simply mistaken as noise. Isotopic distribution patterns created by the use of enhanced universal but random incorporations, such as 5 or 95% ^13^C, allow automated determination of the number of carbons in a metabolite, which as shown in Figure 2, reduces the number of possible formulae (Stupp et al., 2013), and makes identification easier.

In the initial experimental demonstration of IROA, we used *C. elegans* and developed new strategies for isotopic labeling of this model organism (Stupp et al., 2013). The goal was to demonstrate the overall technical approach and to minimize or eliminate biological variation. Therefore, two populations of worms were grown to young adults on a diet of *E. coli* that was grown on either 5 or 95% ^13^C glucose as the primary carbon source. In order to examine the reproducibility of the method, the 5% ^13^C flask was split into 4 experimental replicates and heat shocked at 33°C for 30 min, while the 95% ^13^C control flask was kept at room temperature, as illustrated in Figure 1. The control was then split into 4, combined with the heat shock flasks, and separated into supernatant (exometabolome) and worm pellets (endometabolome). The processed extracts were then analyzed on a high mass-resolution Q-Exactive Orbitrap mass spectrometer (Thermo Scientific), and MS/MS was used to confirm the identity select compounds. For example, we found that purines were down-regulated in the heat shocked endometabolome (Stupp et al., 2013), consistent with literature (Lindquist, 1986; Richter et al., 2010; Morimoto, 2011).

**FIGURE 3:**
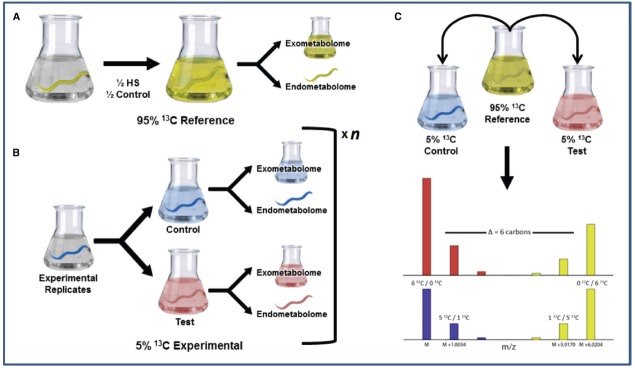
**Experimental design to compensate for isotopic effects. (A)** In this experiment, the 95% ^13^C population is mixed with replicates of both experimental and control groups and serves as an internal ^13^C metabolic reference. **(B)** Both test and control populations are then labeled with 5% ^13^C. **(C)** The 95% ^13^C reference is combined with each 5% test and control in equal concentrations. Such a design allows the 95%, which exhibits the largest isotopic effect (Stupp et al., 2013) to serve as a reference standard by which the 5% control and test metabolites are compared. Figure from the Ph.D. dissertation of Stupp (2014).

It is often difficult or impossible to achieve complete isotopic labeling. In the case of *C. elegans*, not all potential carbon sources could be eliminated. This resulted in a dilution of the planned ^13^C labeling. Assuming the samples were prepared and handled identically, these dilution effects were acceptable because the general patterns still remained and the same material was used as an internal reference for all samples. The dilution/incorporation of the ^13^C in metabolites differed across metabolite features. In the 95% ^13^C channel, we experimentally measured incorporation ranges from about 80 to 98% ^13^C enrichment (Stupp et al., 2013). This range can be reduced by eliminating ^12^C sources of unlabeled nutrients, yet it cannot be totally eliminated, as there will always likely be some enzymatic isotope effects. The concern is whether the experimental study (e.g., heat shock) or the isotope effect is causing an observed change between the 5 and 95% ^13^C channels. For many studies, variation in labeling or isotope effects can be tolerated, because the same reference material is added to all samples. However, if isotope effects are determined to be a problem for quantification, it is possible to correct for these with a modified protocol, as shown in Figure 3. In this case, the 95% ^13^C group is now more appropriately termed a “reference” (Figure 3A), and the 5% ^13^C group is split into two populations, the experiment (e.g., heat shock) and a control (Figure 3B). The 95% reference material is then added to each of the 5% flasks (Figure 3C), and the relative response of each 5% channel compared to the same 95% reference will separate isotope effects from the response to the experimental perturbation.

Isotopic ratio outlier analysis experiments allow for the relative quantification of hundreds to thousands of features, but like all MS-based experiments, exact compound identification remains a challenge. Standardization of LC conditions and calibration with compound libraries can help significantly, but even with known molecular formulae and retention times, a large fraction of features cannot be identified without additional information.

## Nuclear Magnetic Resonance

Sensitivity is the major limitation of NMR in metabolomics and natural products studies, especially with ^13^C detection. The frequency of a resonance transition in NMR is equal to the product of the gyromagnetic ratio and the magnetic field ω_0_ = – *γ* * *B*_0_. A ^1^H nucleus in a 14.1 T magnet has a frequency of 600 MHz, and a ^13^C in the same magnetic field resonates at 150 MHz. The energies of these NMR transitions are approximately five orders of magnitude lower than thermal energy at room temperature. The result is extremely low starting Boltzmann polarization, with only 1 in over 65,000 of the ^1^H nuclei contributing to the signal. It is even worse for ^13^C (1 in 260,000). When this poor starting point is combined with the 1.1% natural abundance of ^13^C, it is not surprising that ^13^C is not typically used in metabolomics or natural products studies.

There are several ways to improve the situation with NMR sensitivity: use higher magnetic fields (costly; Fu et al., 2005); reduce the sample temperature (impractical for biological applications); dynamic nuclear polarization (Ardenkjaer-Larsen et al., 2003; Sze et al., 2012); improved NMR probes (Ramaswamy et al., 2013b); and isotopic labeling. The use of optimized NMR probes is a cost-effective way to improve sensitivity (Olson et al., 1995; Brey et al., 2006). Cryogenic probes with coils constructed from high-temperature superconducting (HTS) materials are especially sensitive and have been recently reviewed (Ramaswamy et al., 2013b). For the studies outlined below, we have used a specialized 1.5-mm cryogenic ^13^C-optimized probe made from HTS material and a sample volume of 40 μL (Ramaswamy et al., 2013a). Small volume probes are desirable for mass-limited samples, but for many ^13^C metabolomics applications, outstanding results can be obtained with commercially available 5-mm ^13^C cryogenic probes.

### Natural Abundance ^13^C

Although the natural abundance of ^13^C is only 1.1%, it is still possible to obtain useful information for compound identification in metabolomics experiments if the probe and acquisition parameters are optimal. In fact, there are several advantages of ^13^C NMR at natural abundance. The resonances are narrow singlets; this significantly reduces resonance overlap and improves the ability to analyze dense spectral regions. In addition, spectral overlap is further reduced by the large spectral width (>200 ppm). Because organic molecules are carbon-based, ^13^C NMR gives a unique insight into the backbone structure rather than the periphery, as detected with ^1^H NMR. Unlike ^1^H-based methods, ^13^C NMR can detect quaternary carbons. With isotopic labeling the ^13^C signal is enhanced; however, isotopic labeling results in loss of the narrow singlets making the ^13^C spectrum resemble the ^1^H spectrum with all of its complications. In addition, isotopic labeling is not always possible.

**FIGURE 4:**
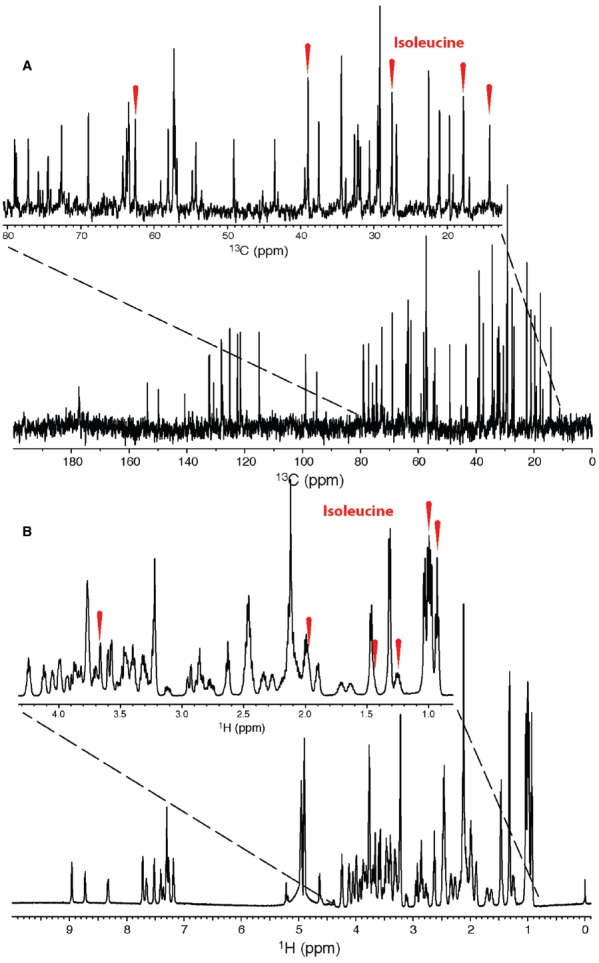
**1D ^13^C (A) and ^1^H (B) NMR spectra of a metabolic mixture.** Due to the ^13^C spectral dispersion and narrow peaks, metabolite resonances such as those belonging to isoleucine (peaks indicated by red ticks), can be easily identified. Resonances in the corresponding 1D ^1^H spectrum often overlap due to coupling and small spectral width, resulting in difficult analysis. Figure used with permission from Ramaswamy et al. (2013a).

Our specialized ^13^C probe (Ramaswamy et al., 2013a) allows for efficient collection of ^13^C 1D data at natural abundance. Figure 4 shows ^1^H and ^13^C NMR spectra of the same sample, which contains a mixture of 20 standard metabolites. The primary spectroscopic advantages of ^13^C NMR are easily seen: large chemical shift dispersion and narrow peaks. These advantages translate to improved metabolomics studies of mixtures (Clendinen et al., 2014). The basic strategy that we developed using just 1D ^13^C and ^1^H NMR data was to use statistical correlations to obtain 2D ^13^C–^13^C and ^13^C–^1^H correlation maps.

**FIGURE 5:**
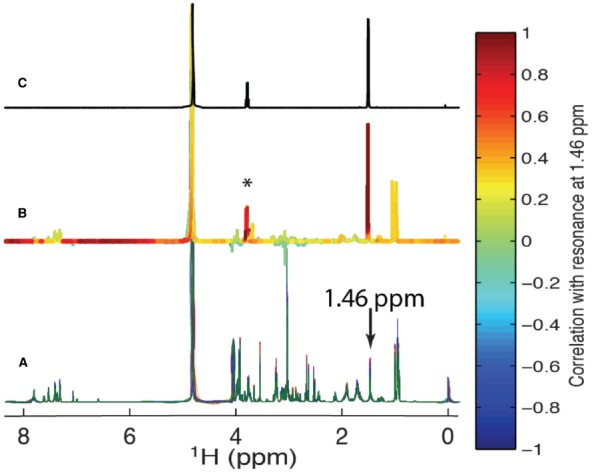
**Statistical total correlation spectroscopy (STOCSY; Cloarec et al., 2005).** In **(A)** we simulated 100 different spectra from a mixture of 20 common metabolites obtained from the BMRB (Ulrich et al., 2008). The concentrations of the metabolites were randomly adjusted in each spectrum. The STOCSY spectrum **(B)** was obtained by correlating all points in the spectra with the alanine methyl resonance at 1.46 ppm. The peak with a high correlation (*) is the alanine α-^1^H. The reference spectrum for alanine from the BMRB is shown in **(C)**.

**FIGURE 6:**
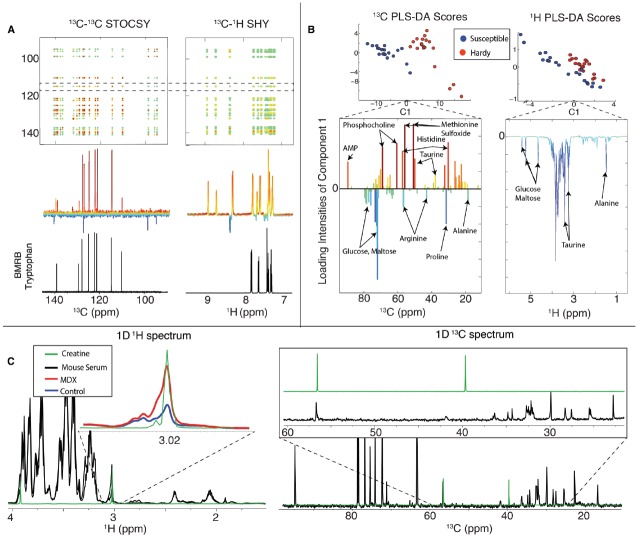
**Summary of natural abundance ^13^C NMR metabolomics**. In **(A)** we show 2D STOCSY and SHY correlation maps that were produced from the same mixture of 20 common metabolites that are shown in Figure 4. We made two groups with five replicates each that had variation introduced by individual pipetting. The 2D ^13^C–^13^C STOCSY spectrum in (A) is the ^13^C statistical correlation map from all the ^13^C 1D NMR data. The 2D ^13^C–^1^H SHY is the statistical correlation map between ^13^C and ^1^H 1D NMR data. In **(B)** we present a summary of a PLS-DA of fruit flies that were either cold susceptible (blue) or cold hardy (red). The left side shows the PLS-DA using ^13^C and the right side using ^1^H data. The improved ^13^C spectral resolution greatly improves the performance in multivariate analyses, such as PLS-DA. In **(C)** we obtained both ^1^H (left) and ^13^C 1D NMR data from mice with a mutation in the *mdx* gene, which causes Duchenne muscular dystrophy in humans. The resonances around 3.02 ppm in the ^1^H spectrum were consistent with creatine (green), but inspection of the ^13^C 1D spectra on the right shows no evidence for the corresponding ^13^C resonances, indicating that creatine is not the major component at 3.02 ppm. Thus, ^13^C data can help prevent misidentifications of metabolites. This figure was adapted from Clendinen et al. (2014), where more details about these studies can be found.

Statistical correlations of NMR or other types of data are extremely useful in metabolomics and natural products studies. There are several variations, but the simplest conceptually is statistical total correlation spectroscopy (STOCSY; Cloarec et al., 2005). We illustrate STOCSY here with Figure 5, in which we used 20 common metabolites from the biological magnetic resonance data bank (BMRB; Biological Magnetic Resonance Data Bank: Ulrich et al., 2008) to simulate 100 different spectra with randomly assigned concentrations of each metabolite (Figure 5A). The key concept underlying STOCSY is that all of the resonances of a given metabolite are perfectly correlated; that is, they will all change intensity in proportion to the concentration of the molecule. This is shown in Figure 5B where we show all correlations to the methyl peak of alanine, which in this case is considered the “driver” peak. When overlap is not severe, STOCSY will result in highly correlated peaks (red in Figure 5B) that are in the same molecule. Compounds from a common biosynthetic pathway can also show weaker correlations. The STOCSY in Figure 5B is one-dimensional, because it is only correlations to the alanine methyl. Two-dimensional STOCSYs (e.g., Figure 6A) can be made by correlating all resonances; these look similar to 2D TOCSY spectra, which correlate resonances spectroscopically rather than statistically. The difference is that STOCSY will also lead to correlations of resonances that are not J-coupled. For example, all of the peaks in phenylalanine will have STOCSY correlations but the aromatic and aliphatic resonances will not be correlated in TOCSY. A more extensive review of different versions of STOCSY has recently been published (Robinette et al., 2013). Another statistical tool that is useful is statistical heterospectroscopy or SHY (Crockford et al., 2006). SHY is essentially identical to STOCSY except that the correlations are between different types of datasets. The original demonstration of SHY was to correlate NMR and ultra performance liquid chromatography (UPLC)-MS (Ultra Performance Liquid Chromatography-MS) datasets, but other types of quantitative data can be analyzed using the same approach. One of the challenges with SHY is to find the proper scaling and normalization for the different datasets. In the example below, we used SHY to correlate ^1^H and ^13^C 1D data, which produces statistical maps that are similar to spectroscopic 2D HSQC-TOCSY data.

These statistical correlation maps were very useful in that they allowed us to generate peak lists that could be used to query databases of known compounds. When we compared the results of using ^1^H data alone with ^13^C, we were able to correctly match more metabolites to the BMRB database. We also investigated S/N limits of this approach and found that 60 nmol in 40 μL was about the lower limit with the NMR probe (Ramaswamy et al., 2013a) and parameters we used for data collection (∼2 h per spectrum). We discuss factors influencing NMR sensitivity below.

Multivariate analysis is routinely used in metabolomics. The simplest and most common approach is principal component analysis (PCA). The goal of PCA is to represent a high-dimensionality dataset in just a few dimensions that capture most of the variance. For example, in NMR data, each data point in a spectrum can be considered a dimension, meaning that for even 1D NMR studies the dimensionality is often as high as 64 or 128 k. Using PCA the data are rotated to a new coordinate system, which represents the variance of the data, ordered from highest to lowest. Thus, the first principal component (PC1) will be the axis with the greatest variance in the data, followed by PC2, PC3, etc. The output of a PCA is a scores plot, which is often a two-dimensional representation (e.g., PC1 vs. PC2) where every point is a sample in the study. An example of a simple PCA scores plot is shown in the inset of Figure 8 below, in which heat-shocked *C. elegans* (red triangles) are separated along PC1 from the controls (blue circles). Loadings plots indicate the specific features (e.g., NMR resonances or MS m/z values) that are responsible for the group separation represented by the scores plot.

Principal component analysis is an unsupervised technique, which means that the algorithm does not use information about sample groups (e.g., heat-shocked vs. controls). This is in contrast to supervised methods that use information about the origin of the samples. For example, in Figure 6B we show partial least squares-discriminant analysis (PLS-DA), a supervised method that includes in the algorithm knowledge of the groups (in Figure 6B cold hardy vs. cold susceptible fruit flies). Supervised methods are widely used and can be very useful, but they also must be used with care, because they are starting with a bias that the groups are meaningful to any differences that might be found.

We found that using ^13^C NMR at natural abundance led to improved performance in both PCA and PLS-DA. We compared the group separation and loadings using ^13^C NMR over 1D ^1^H alone. This improved performance follows from the greater spectral dispersion of ^13^C resonances when compared with ^1^H, as shown in Figure 4.

By using 1D ^13^C NMR, metabolite identification can be more robust when combined with ^1^H data. For example, we compared data from *mdx* mice (models for Duchenne muscular dystrophy) with control and found a peak in the ^1^H NMR spectra at 3.02 ppm that is normally ascribed to creatine. However, the 1D ^13^C NMR spectra did not contain the corresponding ^13^C resonance, which allowed us to rule out creatine as being the largest contributor to the ^1^H resonance (Figure 6C). Thus, 1D ^13^C NMR was able to prevent the misidentification of a metabolite. Our study showed that 1D ^13^C NMR global metabolomics at natural abundance is feasible and also yields (1) improved metabolite identification through the use of better peak lists resulting from reduced peak overlap (Figure 6A), (2) improved multivariate statistical analysis and therefore better group separation and more informative loadings plots (Figure 6B), and (3) additional important data, which can prevent the misidentification of metabolites (Figure 6C; Clendinen et al., 2014).

The major limitation to ^13^C NMR at natural abundance is sensitivity. The ^13^C 1D spectra required for the study summarized above required about 2 h each with very rapid recycling rates that attenuated resonances with long T_1_ relaxation times like quaternary carbons. The small volume probe that we used is ideal for mass-limited samples and provides excellent results for samples that can be concentrated. However, it is not ideal for samples with limited concentrations. The overall design of the NMR probe and sample size is critical in optimizing performance, and there are many factors that need to be considered. Two sample scenarios need to be taken into account: mass limited or concentration limited. When samples are mass limited, it is best to use the smallest volume probe and sample tube, because mass sensitivity is inversely proportional to the diameter of a sample (Olson et al., 1995). This is the situation with many natural product studies (e.g., Srinivasan et al., 2008; Dalisay et al., 2009; Molinski, 2009; Dalisay and Molinski, 2010). However, if the sample is not mass limited or if the sample has limited solubility, a larger volume probe will perform better. Another factor is the effect of salt, which can seriously degrade the performance of NMR probes, especially cryogenic probes (Kovacs et al., 2005). A smaller volume tube (Voehler et al., 2006) or a rectangular geometry (de Swiet, 2005) will improve cryogenic probe performance in salt. Salt loss is also dependent on frequency, with higher frequencies showing more severe loss than lower (Horiuchi et al., 2005). Therefore, an additional advantage of ^13^C detection is that it is less salt dependent than ^1^H detection. The small volume ^13^C probe that we used in the studies described here is optimal for mass limited samples. For example, using the 60 nmol of material mentioned above, we would expect between 2 and 3 times greater S/N from that sample in 40 μL using our customized HTS probe than the same 60 nmol in 600 μL using a commercial 5-mm cryogenic ^13^C-optimized probe (Ramaswamy et al., 2013a). However, as previously described (Clendinen et al., 2014), if the sample quantity is not limited, the same concentration of sample in a 5-mm ^13^C-optimized probe would produce about seven times greater S/N than we were able to measure in our reduced volume probe.

### ^13^C Isotopic Enrichment

To improve ^13^C sensitivity in NMR, the most straightforward approach is isotopic labeling. Although labeling adds cost to the sample preparation and is not always possible, it can greatly expand the utility of NMR-based metabolomics and natural products studies. The benefit of labeling goes beyond the obvious advantage of increasing the number of ^13^C nuclei beyond natural abundance. The method we describe below is based on the INADEQUATE experiment, which provides networks of carbons from correlations of directly bonded ^13^C atoms. INADEQUATE is one of the most powerful 2D NMR experiments for the identification of unknown compounds, but it is rarely used because of its insensitivity. An example of a nice alternate approach is the use of ^1^H detected ^13^C–^13^C TOCSY with the TOCCATA (TOCSY Customized Carbon Trace Archive) database developed by the Brüschweiler laboratory (Bingol et al., 2012). TOCCATA is a metabolomics NMR database adapted from the BMRB (Ulrich et al., 2008) and the human metabolome database (HMDB; Wishart et al., 2013) and contains over 400 compounds. The ^13^C–^13^C TOCSY, when used with TOCCATA, has yielded impressively accurate metabolite query results when compared to existing ^13^C chemical shift queries online (Bingol et al., 2012). The Brüschweiler laboratory has developed several other approaches using ^13^C NMR for metabolomics mixture analysis (Bingol et al., 2012, 2013, 2014a). The database matching protocol using just ^13^C-HSQC (directly bonded ^13^C–^1^H pairs) is particularly useful for generating potential matches to databases (Bingol et al., 2014b).

### INADEQUATE

Incredible natural abundance double quantum transfer experiment obtains correlations of directly bonded ^13^C networks for unknown compound identification (Bax et al., 1980; Sorensen et al., 1982; Buddrus and Bauer, 1987). However, at natural abundance, very high concentrations of compounds are needed in order for the INADEQUATE experiment to provide any useful information. As stated previously, INADEQUATE reveals the carbon backbone of a molecule by connecting carbon networks through direct bond correlations. Beyond the general problems of ^13^C NMR sensitivity described above, INADEQUATE relies on adjacent ^13^C nuclei, which at natural abundance have a probability of 1 in 8264. However, with 99% ^13^C labeling, the probability of adjacent ^13^C atoms is essentially 100%. Markley and co-workers showed that excellent results on proteins could be obtained using INADEQUATE with 26% ^13^C labeling (Oh et al., 1988). This lower percentage of ^13^C slightly decreases the probability of obtaining two adjacent spins but also decreases longer range couplings and higher order interactions that are observed with 99% ^13^C labeling. A combination of ^13^C isotopic labeling, as well as better ^13^C sensitive HTS probe designs (Ramaswamy et al., 2013a), allows for INADEQUATE NMR to be a useful tool for metabolomics and compound identification (Clendinen et al., 2015).

**FIGURE 7:**
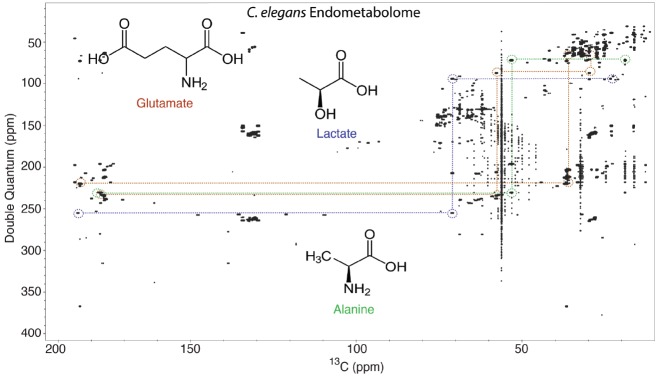
**2D INADEQUATE NMR spectrum of the endometabolome of ^13^C-labeled ***C. elegans***.** The horizontal axis is the ^13^C chemical shift, and the vertical axis is the double quantum chemical shift. Cross-peaks appear on the double quantum axis at the sum of the two interacting ^13^C frequencies. This spectrum has a very large amount of information, and we developed INETA to extract some of this information in a semi-automated way. Details can be found in the original publication (Clendinen et al., 2015), but some of the output of INETA applied to this spectrum is shown in the dashed lines, which highlight INADEQUATE spin systems from glutamate (brown), lactate (blue), and alanine (green) in the endometabolome.

Figure 7 shows an INADEQUATE spectrum collected from the endometabolome of one million *C. elegans* that have been isotopically labeled with 99% ^13^C. Clearly there is a great deal of information in this NMR spectrum, which is comparable in its complexity to a standard ^1^H–^1^H COSY experiment of a complex mixture. Some ^13^C–^13^C networks of metabolites are included in Figure 7 to illustrate the sort of information contained in these spectra. To make this approach useful for metabolomics and large numbers of samples, the process must be automated.

One of the advantages of ^13^C NMR is efficient database matching of known compounds. However, there are no databases of INADEQUATE spectra for metabolites and natural products. In order to construct an *in silico* database, one only needs ^13^C 1D spectra of a known compounds with resonance assignments. For two correlated ^13^C resonances, an INADEQUATE spectrum has two peaks; the horizontal axis gives the ^13^C chemical shifts of each resonance and the vertical double quantum axis is the sum of the two ^13^C chemical shifts (Figure 7). Using this information, we have made an INADEQUATE database from over 1000 reference metabolites in the BMRB. To extract metabolites from spectra like that shown in Figure 7, we have written a software package called INETA (INADEQUATE Network Analysis) that identifies networks in experimental INADEQUATE spectra (Clendinen et al., 2015). The steps of INETA are (1) to peak-pick the spectrum, (2) find all the pairs of peaks that follow the rules of INADEQUATE (double quantum frequency is the sum of the two interacting peaks), (3) match the chemical shifts of all the pairs to make partial or complete ^13^C–^13^C networks, and (4) match the networks to the *in silico* database. This is all described in complete detail in the primary reference (Clendinen et al., 2015). Using INETA, the NMR analyst can also directly analyze networks that did not match known compounds. Unknown compounds can be discovered using traditional natural products approaches of ^13^C network analysis with the addition of LC-MS data, such as IROA, and quantum mechanical calculations of ^13^C chemical shifts (Wang et al., 2009).

**FIGURE 8:**
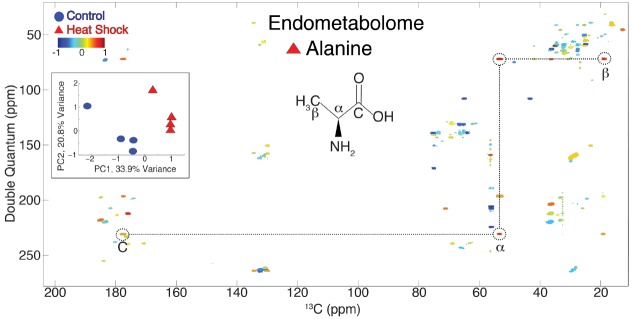
**Principal component analysis scores and loadings plot of control (blue circle) and heat shock (red triangle) for all the INADEQUATE 2D NMR data from the ***C. elegans*** endometabolome.** This 2D NMR PCA was developed for TOCSY spectra but can be applied to any 2D NMR data (Robinette et al., 2011). The PCA scores plot (inset) displays clear separation along PC1. The loadings plot from PC1 is shown in the main spectrum and indicates which resonances are correlated with the control (blue) or heat shock (red). Alanine, for example, strongly correlates with heat shocked animals (Clendinen et al., 2015).

In addition to extracting networks, we are able to analyze multiple INADEQUATE spectra from a metabolomics study using a 2D NMR multivariate analysis method developed previously (Robinette et al., 2011). When networks from INETA are superimposed onto the resulting PCA 2D NMR loadings plots, one can quickly identify metabolites that change in a study (Figure 8).

### Combining NMR and IROA for Compound ID

Isotopic ratio outlier analysis relies on database matching or standard libraries for definitive compound identification, and discrimination of isomers can be difficult or impossible using just MS alone. To identify truly unknown compounds or those not in databases, it is generally necessary to combine LC-MS with NMR (Lambert et al., 2007; Tayyari et al., 2013; Wolfender et al., 2013). The challenge is that NMR sensitivity is much lower than LC-MS sensitivity. A properly selected NMR probe can help. Although many factors influence NMR probe sensitivity, as discussed above, one of the major variables is size: NMR mass sensitivity is roughly proportional to the inverse of the diameter of the coil of the probe. Thus, a small volume probe can help bridge the NMR/LC-MS sensitivity mismatch. Using our 1.5-mm ^13^C-optimized probe, we have been exploring ways to combine the power of NMR in structural determination with LC-MS sensitivity. The overall approach is to collect fractions from an LC-MS run of an IROA experiment. If necessary, several samples could be reinjected to obtain enough material for NMR. Then, using the LC-MS as a guide, we find an IROA fraction of interest that we are not able to identify using libraries or databases. The LC-MS fraction can be dried, and the resulting material can be resuspended in an appropriate NMR solvent. The IROA labeling leads to a mixture of 5 and 95% ^13^C, and for ^13^C NMR the majority of the signal originates from the 95% material. In our preliminary studies, we have been able to collect useful ^13^C 1D as well as various 2D NMR data with five LC-MS injections. The ^13^C chemical shifts, along with other 2D experiments and the molecular formula provided from the IROA LC-MS experiment, are often enough to identify unknowns.

## Conclusion

^13^C-based metabolomics is both useful and practical. Using a combination of isotopic labeling strategies, high-resolution LC-MS instruments, and ^13^C-optimized NMR probes, it is now possible to more efficiently dereplicate complex mixtures through improved database matching and to identify unknown metabolites or natural products of interest. LC-MS techniques such as IROA allow for the detection of thousands of features in an untargeted manner. IROA not only allows for the discrimination of features from the background, but also provides relative quantitation of features and a more accurate estimate of molecular formulae. Natural abundance ^13^C NMR can give nice advantages over exclusively ^1^H-based methods due to narrow peaks that are well-resolved over large spectral widths. Isotopic labeling greatly increases the S/N of ^13^C NMR, especially in ^13^C–^13^C correlation experiments like INADEQUATE. By combining NMR and LC-MS experiments, unknown compounds can be identified.

### Conflict of Interest Statement

Chris Beecher is the inventor and CSO of IROA Technologies. Patent 7,820,963. Beecher (Inventor) “Method for generation and use of isotopic patterns in mass spectral data of simple organisms,” issued October 2010. This is the base IROA patent (main patent and CIPs). Patent 7,820,964. Beecher (Inventor) “Method for generation and use of stable isotope patterns in mass spectral data” issued October 2010. This is the IROA standards patent. The other authors declare that the research was conducted in the absence of any commercial or financial relationships that could be construed as a potential conflict of interest.
